# Patient Engagement and Patient Safety: Are We Missing the Patient in the Center?

**DOI:** 10.7759/cureus.7048

**Published:** 2020-02-19

**Authors:** Muhammad Hasan Abid, Muhammad Mohsin Abid, Salim Surani, Iqbal Ratnani

**Affiliations:** 1 Internal Medicine, Institute for Healthcare Improvement, Boston, USA; 2 Internal Medicine, Jinnah Medical College, Peshawar, PAK; 3 Internal Medicine, Texas A&M Health Science Center, Bryan, USA; 4 Internal Medicine, Corpus Christi Medical Center, Corpus Christi, USA; 5 Internal Medicine, University of North Texas, Dallas, USA; 6 Internal Medicine, Weil Cornell College of Medicine, Houston, USA

**Keywords:** patient-centered care

## Abstract

The global healthcare delivery paradigm shift calls for enhanced strategies to engage patients in delivering safer and high-quality healthcare. There still exists a gap area in a globally accepted measure for the person-centered care. Recent tri-institutional global quality reports from National Academies of Sciences, Engineering, and Medicine (NAESM), World Bank Group, and Lancet Global Health Commission attempted to report the patient engagement measures used globally. We aim to understand the variation in these globally reported patient-centered care measures and highlight the recent proactive strategies to enhance patient engagement to improve patient safety.

I

## Introduction and background

It has been 18 years since Institute of Medicine (now the National Academy of Medicine) in its 2001 seminal report *Crossing the Quality Chasm *recognized person-centered care as a domain of healthcare quality [1]. Person-centered care (also known as patient-centeredness) requires “providing care that is respectful of and responsive to individual patient preferences, needs, and values and ensuring that (these) values guide all clinical decisions” [1]. The National Academies of Sciences, Engineering, and Medicine (NASEM) in its report *Crossing the Global Quality Chasm: Improving the Healthcare Worldwide*, noted the overall experience of patients in healthcare systems as levels of dissatisfaction varying 2.2%-54.3% globally [2]. The Lancet Global Health Commission, in its report *High-Quality Health Systems in the Sustainable Development Goals Era: Time for a Revolution*, revealed that an average of 34% of people in low-income and middle-income countries (LMICs) reported poor user experience when came in contact with their respective healthcare systems [3]. For high-income countries (OECD), an age standardized rate of 81.3 per 100 patients was reported on doctor providing easy-to-understand explanations [4]. It is clear that globally there is an increasing awareness and action among the healthcare stakeholders to improve the patient-centeredness; however, there is ambiguity on what it actually means, or how-to best measure this in a standardized fashion [2]. This has also led to variability in strategies used to collect data on patient-centeredness ranging from patient experience surveys to the patient-reported outcome measures. Patient-centeredness is a critical component of quality healthcare and also strongly linked with the safety of healthcare delivery amongst other dimensions of healthcare quality. Therefore, healthcare organizations and health systems need to develop more applicable, standardized, and effective patient engagement strategies.

## Review

Currently across the healthcare systems worldwide, there is a strong push towards newer model of care that shifts the conversation from “What’s the matter with you?” to “What matters to you?.” Patient and family engagement (‘patient engagement’ for simplicity) is defined as ‘patients, families, their representatives, and health professionals working in active partnership at various levels across the healthcare system (direct care, organizational design and governance, and policy making) to improve health and healthcare’ [5]. It merges patient activation (an individual’s knowledge, skills, ability and willingness to manage his/her own health and care) with interventions designed to increase activation and promote positive patient behavior [5].

Idea of patient engagement can be traced back to the traditional model of medical practice with limited patient engagement leading to health outcomes (good and bad) that are co-produced, synonymous with other service providing industries and fields [6-7]. Therefore, healthcare service is co-created by healthcare professionals in relationship with one another and with people seeking help to restore or maintain health for themselves and their families [6]. The Joint Commission directed that healthcare organizations "encourage patient’s active involvement in their own care as a patient safety strategy" as a National Patient Safety Goal in 2007 [8]. Patient engagement forms the core of Institute for Healthcare Improvement’s (IHI) framework for safe, reliable, and effective care [9]. Moreover, patient engagement is also one of the strategies to achieve the IHI triple aim of improved health outcomes, better patient care, and lower costs [10]. Over the last decade, patient engagement has been employed in the context of shared decision-making, encouraging patients to raise concerns, and maintaining a safety culture that encourages patient empowerment facilitating an increased diversity and maturity of the patient engagement literature.

Carman et al. proposed a multidimensional framework that conceptualized three critical aspects of patient engagement in health and healthcare across the continuum of the engagement (ranging from consultation and involvement to partnership and shared leadership), at different levels of engagement (from direct care interaction to organizational design and governance and policy making) and affected by multiple factors [5]. An example of organizational design and governance level of patient engagement across the higher end of continuum of engagement will be the work at the Dana-Farber Cancer Institute, where patients and family members have participated as decision-making members in continuous quality improvement teams, taken part in hiring decisions, and developed and provided staff training [11].

Recent studies have examined patient preferences for involvement, and emergence of novel tools to promote engagement and revamped care models that activate patients [12-13]. Agency for Healthcare Research and Quality’s Guide to Improving Patient Safety in Primary Care Settings by Engaging Patients and Families provides resources for providers to partner with patients to improve engagement in ways that can impact safety [14]. The Promoting Respect and Ongoing Safety Through Patient Engagement Communication and Technology (PROSPECT) study concluded that implementation of a structured team communication and patient engagement program in the ICU was associated with a 30% reduction in adverse events and improved patient and care partner satisfaction [12]. On the contrary, the Patient Report and Action for a Safe Environment (PRASE) cluster randomized controlled trial found that surveying inpatients about their safety experiences had no impact on a global measure of safety [13]. Interventions in the form of checklists for bedside family-centered rounds for hospitalized children, enable parents to engage at all aspects of the care plan, improve the quality of care, and improve the patient safety from the parents’ perspectives [15]. Notably, the OpenNotes project was demonstrated to help engage patients as safety partners without apparent negative consequences for clinician workflow or patient-clinician relationships [16]. Sawhney et al. found that patients and families wanted Internet-based, telephone, mailed, smartphone application-based, and face-to-face options to report their safety concerns, suggesting that facilitating patient engagement via multiple health information technology (IT) enabled approaches may be effective for engaging patients in safety by gathering safety concerns and also activating patients in their care [17]. Apart from the patient factors influencing the engagement (e.g., patient’s health literacy), health IT has the capability to customize the degree of engagement, however, additional work is required to emphasize development and testing of health IT approaches that work in diverse real-world settings. Nonetheless, the verdict is out with recent work on paying for relationship rather only the performance [18]; careful and kind care requiring unhurried conversations with patients and families [19]; and preserving the patient-physician relationship by balancing the medicine as humanitarian profession and healthcare as a competitive business [20].

## Conclusions

To conclude, patient engagement is an ever-expanding area as increasingly organizations look into involving patients and families in patient safety, quality improvement, and health system design. The recent work has demonstrated significant benefits of system redesign in effectively engaging the patients and families as they navigate through the complex healthcare systems to avoid the safety issues and medical errors. It is now an uphill action item for the healthcare leaders to harvest the best enabling practices across the healthcare and other industries to leverage the technological platforms in implementing the most value adding practices linked to operationally and clinically relevant patient engagement measures which will impact the patient centeredness and also improve the safety of the healthcare being delivered.
